# Dietary pollen and carbohydrate sources influence longevity, physiology, and gut microbiota in honey bee (*Apis mellifera*)

**DOI:** 10.1038/s41598-026-48586-0

**Published:** 2026-04-16

**Authors:** Arezoo Najarpoor, Saeed Mohamadzade Namin, Sampat Ghosh, Chuleui Jung

**Affiliations:** 1 Department of Plant Medicals, Gyeongkuk National University, Andong, Republic of Korea; 2 Department of Horticulture, College of Agricultural Sciences, Oregon State University, Corvallis, OR USA; 3 Department of Life Science, Sardar Patel University, Balaghat, Madhya Pradesh India; 4 Agricultural Science and Technology Institute, Gyeongkuk National University, Andong, Republic of Korea

**Keywords:** Pollinator, Nutrition, Carbohydrate source, Pollen, Gut microbiota, Beekeeping, Ecology, Ecology, Physiology, Zoology

## Abstract

The Western honey bee (*Apis mellifera*) is essential for crop pollination and ecosystem stability and production for beekeepers as well, yet rising colony losses are a major concern. Poor nutrition is a key stressor, prompting beekeepers to supplement carbohydrates and proteins during nectar and pollen shortages. This study was conducted under controlled laboratory cage conditions to evaluate the interaction effects of two carbohydrate sources, regular corn syrup (rich in maltose) and sucrose syrup; and three pollen types (rapeseed, kiwi, and mixed pollen) on honey bee longevity and physiological performance. Bees fed corn syrup lived longer and developed larger hypopharyngeal glands (HPG) than those fed sucrose syrup. Among pollen treatments, mixed pollen produced the most favorable outcomes, including increased survival, larger HPG acini, and higher vitellogenin levels. The combination of corn syrup and mixed pollen (CS–MP) yielded the longest lifespan, and lowest food consumption, indicating improved nutritional efficiency. Gut microbiota composition varied with diet: RP and KP diets showed higher relative abundances of *Sondgrassella* and *Gilliamella* compared with MP whereas the mixed pollen diet promoted a higher relative abundance of *Lactobacillus*. These results demonstrate that optimizing carbohydrate and pollen combinations can significantly enhance honey bee health, longevity, and colony resilience, providing practical insights for nutritional management in apiculture.

**Supplementary Information:**

The online version contains supplementary material available at 10.1038/s41598-026-48586-0.

## Introduction

The European honey bee (*Apis mellifera*) is a cornerstone of global agriculture, playing a vital role in pollinating a wide variety of crops^1^. Beyond agriculture, honey bees support biodiversity by facilitating plant reproduction and sustaining natural ecosystems^2^. Despite their importance, managed honey bee colonies have experienced alarmingly high annual losses over the past three decades, particularly during winter months^3^. These colony losses are driven by multiple factors, including parasites, agrochemical exposure, and inadequate nutrition^4,5^. Among these stressors, nutrition plays a key role in honey bee health. Poor diets weaken immune systems^6^, increase disease susceptibility^7,8^, and exacerbate pesticide sensitivity^9^. Additionally, nutritional deficiencies accelerate the transition of worker bees from in-hive tasks to foraging, a shift linked to reduced longevity^10^. Because worker lifespan is closely linked to the timing of foraging initiation, bees that begin foraging earlier typically exhibit shorter lifespans^11^. The natural diet of honey bees consists of nectar, their primary carbohydrate source containing trace amounts of amino acids and lipids^12,13^, and pollen, which provides proteins, essential amino acids, lipids, starch, sterols, vitamins, and minerals^14^. A diverse floral diet is necessary to meet bee’s nutritional needs, as pollen nutritional composition varies by plant species^15^. However, modern intensive agriculture has reduced floral diversity, leading to nutritional deficiencies^16,17^. Malnutrition is especially problematic during summer or when colonies are placed near monocultures, which offer low-quality pollen and nectar^18^. Limited protein intake reduces brood rearing and shortens worker lifespans, highlighting the essential role of pollen nutrition in colony sustainability. To mitigate nutritional stress, beekeepers often provide artificial diets supplemented with proteins, fats, vitamins, and minerals, especially when natural pollen is scarce or of poor quality^19,20^. Artificial feeding studies show that diet composition directly influences both individual bee physiology and overall colony health^21^ and strength^22,23^. Diets enriched with natural pollen have been shown to provide superior benefits, including enhanced immune function^24^, improved resilience to infectious diseases and environmental stressors^24,25^, and better overall colony performance^22^. This highlights the critical role of high-quality protein sources in maintaining honey bee health and longevity^26,27^. Among pollen sources, rapeseed^28^ and kiwi^29^ are considered highly nutritious for honey bees, with protein content reaching approximately 26%. These pollen types not only offer high protein concentrations but also contain essential amino acids that support bee growth and immune system function^27^. Many studies have explored the taxonomic composition and functional roles of the honey bee gut microbiome with a particular focus on the worker bees^30^. Gut bacteria play a symbiotic role by supporting carbohydrate metabolism^31,32^, stimulating the immune system^33,34^, protecting bees from pathogens through competitive inhibition^35^, production of antifungal metabolites^36^, and enhancing detoxification^37^. Studies showed that the composition of supplementary food influences the gut microbiome abundance and community structure^38^ and the diversity of pollen in the diet plays a crucial role in shaping the honey bee gut microbiome^39^. Bees fed a poly floral diet showed higher levels of core symbiotic bacteria compared with those consuming a mono floral diet^40^. Furthermore, diets enriched with probiotics, such as pollen substitutes, can reshape microbial communities by boosting beneficial bacteria and enhancing overall bee health^41^.

Our recent trial on identifying the best carbohydrate source for honey bees demonstrated the superior performance of corn syrup and sucrose syrup over other artificial feeding options, significantly enhancing honey bee longevity and physiological parameters in caged bees^42^. Building on these findings, this study aims to evaluate the combined effects of carbohydrate and protein sources on honey bee health, physiology and gut microbiome composition. Specifically, we investigate the impact of rapeseed pollen, kiwi pollen, and a 50:50 mixed pollen blend in combination with two carbohydrate sources, corn syrup and sucrose syrup, to determine the optimal nutritional combination for honey bees. Since extending individual bee lifespan alone is insufficient for long-term colony success, we also assess key physiological indicators, including hypopharyngeal gland (HPG) size and vitellogenin (*vg*) expression, both of which are linked to nurse bee health and performance^43,44^. Additionally, as diet influences gut microbiome composition, which plays a critical role in digestion, nutrient absorption, pathogen defense, and detoxification^45,46^, we incorporate microbiome analysis into our evaluation. We hypothesize that mixed pollen will have the most beneficial impact on honey bee health compared to mono floral pollen sources.

## Results

### Assessing honey bee lifespan and food consumption

**Fig. 1:**
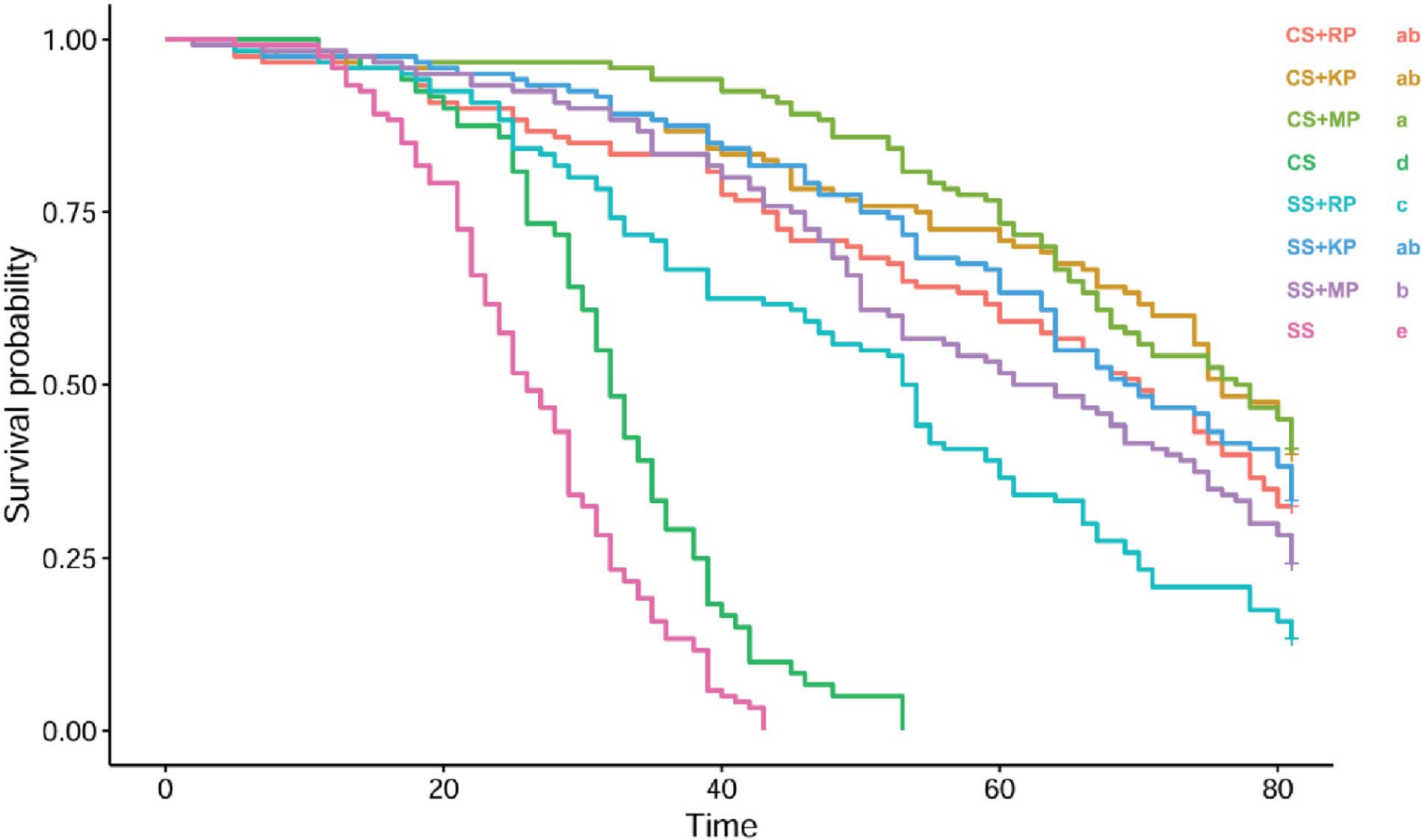
Kaplan – Meier survival curve of caged honey bees fed different carbohydrates and pollen diets. Survival differences among treatments were evaluated using the log-rank test. Different lowercase letters indicate significant differences between treatments based on pairwise log-rank comparisons (*p* < 0.05). SS: sugar syrup; CS: corn syrup; RP: rapeseed pollen; KP: kiwi pollen; MP: mixed pollen.

Kaplan-Meier survival analysis indicated that the treatment groups receiving CS as a carbohydrate source had higher overall survival. The CS-MP group exhibited the highest survival rate, followed by CS-KP, CS-RP, and SS-KP. The survival rate of bees fed with SS-MP and SS-RP were significantly lower than CS-MP group. In the groups that received only carbohydrate, the survival of CS group was significantly higher than SS treated group (*p* < 0.001) (Fig. 1). The mean longevity of bees fed the CS-MP diet was significantly greater than of bees consuming SS-MP diet. No significant difference in longevity was observed between the CS-KP and SS-KP treatments. Notably, bees fed CS alone exhibited significantly greater longevity than those fed SS alone (Fig. 1) .

Honey bee longevity was significantly affected by carbohydrate source (two-way ANOVA, F = 9.27, df = 1, *p* < 0.05, η² = 0.188), with honey bees fed CS exhibiting higher longevity in comparison with those receiving SS as a carbohydrate source. Additionally, pollen type had a significant effect on honey bee longevity (two-way ANOVA, F = 70.52, df = 3, *p* < 0.001, η² = 0.841), with groups receiving MP and KP exhibiting significantly higher longevity than RP fed groups. However, the interaction between carbohydrate source and pollen type was not significant (two-way ANOVA, df = 3, F = 0.961, *p* = 0.421, η² = 0.067).

**Fig. 2:**
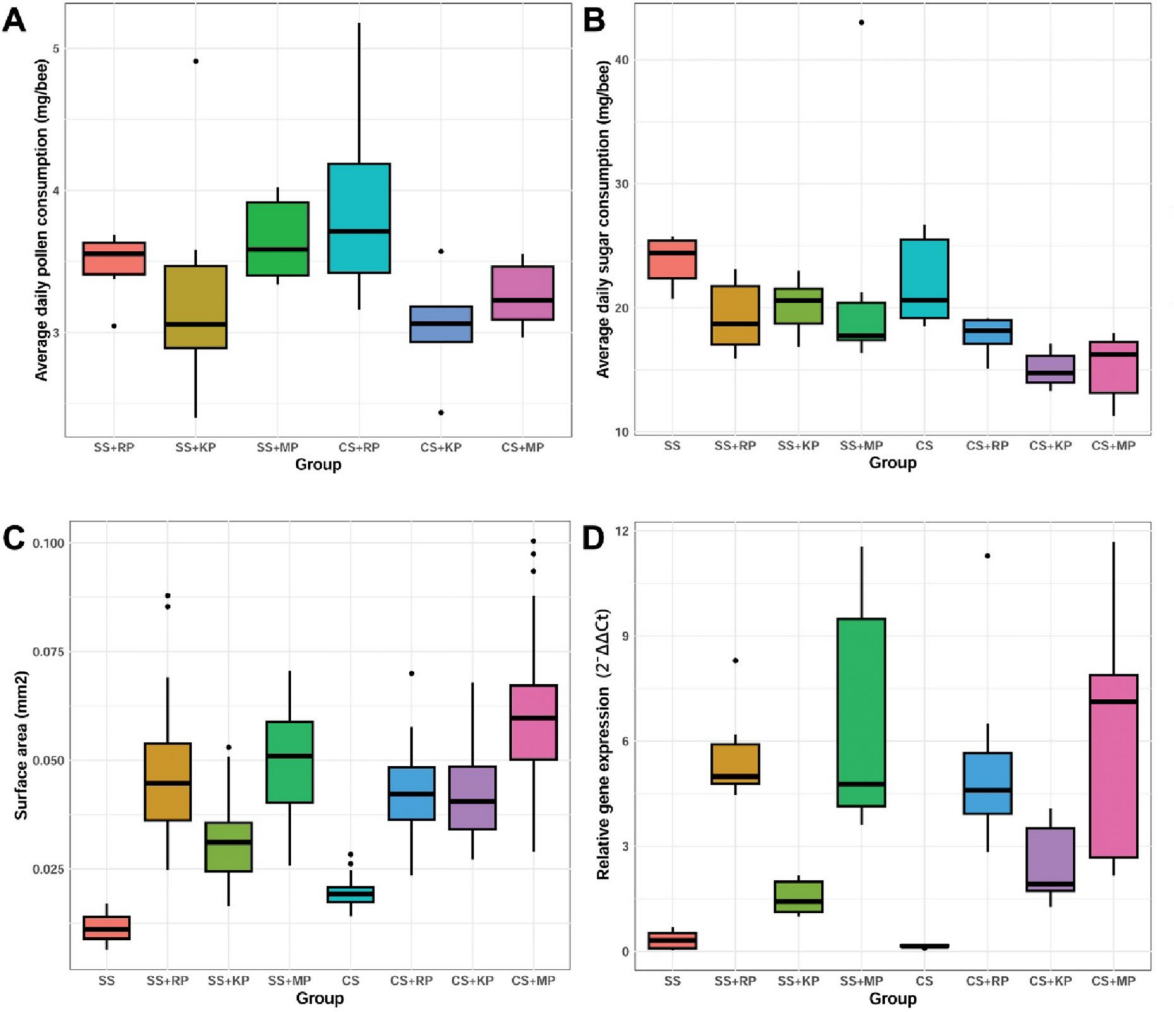
Mean daily consumption of pollen (**A**) and carbohydrate source (**B**) (mg/bee), Hypopharyngeal gland acini surface area measured in 7-day-old caged honey bees (**C**) and relative expression of the vitellogenin (*vg*) gene in 7-day-old caged honey bees (**D**) fed different carbohydrates and pollen diets. Bars represent mean ± SE. Different letters indicate significant differences among treatment groups (*p* < 0.05). SS: sugar syrup; CS: corn syrup; RP: rapeseed pollen; KP: kiwi pollen; MP: mixed pollen.

Regarding pollen consumption, the results showed that the main effect of carbohydrate source was not significant (ART ANOVA, df = 1, F = 0.108, *p* = 0.744), indicating that carbohydrate type did not influence pollen consumption. However, the main effect of pollen type was significant (ART ANOVA, df = 2, F = 4.473, *p* = 0.019), suggesting that pollen type significantly affected consumption. The interaction between carbohydrate source and pollen type was not significant (ART ANOVA, df = 2, F = 2.194, *p* = 0.129) (Fig. 2A). The highest pollen consumption was observed in the RP-fed group, whereas the lowest consumption was recorded in the KP-fed group. Tukey’s post hoc test indicated that pollen consumption in the RP-fed group was significantly higher than in the KP-fed group (t = − 2.955, *p* = 0.016).

In addition, the results indicate that the average SS consumption was 0.036 µL/bee which was significantly higher than 0.025 µL/bee consumption in CS treatment groups (two-way ANOVA, *P* < 0.001). CS-KP and CS-MP treated groups had the lowest carbohydrate consumption among all treated groups (0.022 µL/bee). However, the interaction effect between carbohydrate source and pollen type was not significant (F = 0.915, *p* = 0.442, η² = 0.064), indicating that the effect of pollen type on sugar consumption did not depend on the carbohydrate source (Fig. 2B).

### Measuring hypopharyngeal gland acinus size

The results indicated that the effect of pollen type on HPG size was significant (ART ANOVA, df = 3, F = 290.926, *p* < 0.001). Among pollen types, the group received MP resulted in the largest HPG size (0.055 mm^2^), followed by RP (0.044 mm^2^), and KP (0.036 mm^2^) and no pollen groups (0.015 mm^2^). Also the carbohydrate source had a significant effect on HPG size (ART ANOVA, df = 1, F = 52.355, *p* < 0.001,), with bees fed CS having statistically larger HPGs compared to those fed SS. Additionally, the interaction between carbohydrate source and pollen type was also significant (ART ANOVA, df = 3, F = 8.085, *p* < 0.001), indicating that the combined effects of pollen and carbohydrate source influenced HPG size. The results demonstrated that CS-MP (0.060 mm^2^) and SS-MP group (0.050 mm^2^) had the largest HPG size compare to all treated groups (Fig. 2C).

### Gene expression analysis

The relative expression of *vg* gene in worker bees varied significantly across different treatment groups (Fig. 2D). The highest *vg* expression was observed in bees fed CS-MP followed closely by those receiving SS-MP. In contrast, bees that received only carbohydrate sources (SS or CS) exhibited the lowest *vg* expression levels. Data analysis reviled that pollen type had a significant main effect on *vg* expression (two-way ANOVA, df = 3, F = 11.519, *p* < 0.001, η² = 0.684), whereas the carbohydrate source had no significant effect (two-way ANOVA, df = 1, F = 0.025, *p* = 0.876, η² = 0.002). The interaction between carbohydrate source and pollen type was also not significant (two-way ANOVA, df = 3, F = 0.164, *p* = 0.919, η² = 0.030). Overall, no significant differences were observed between the RP and MP groups. However, significant differences were found between RP and KP (*p* = 0.050).

### Gut microbiome analysis

**Fig. 3:**
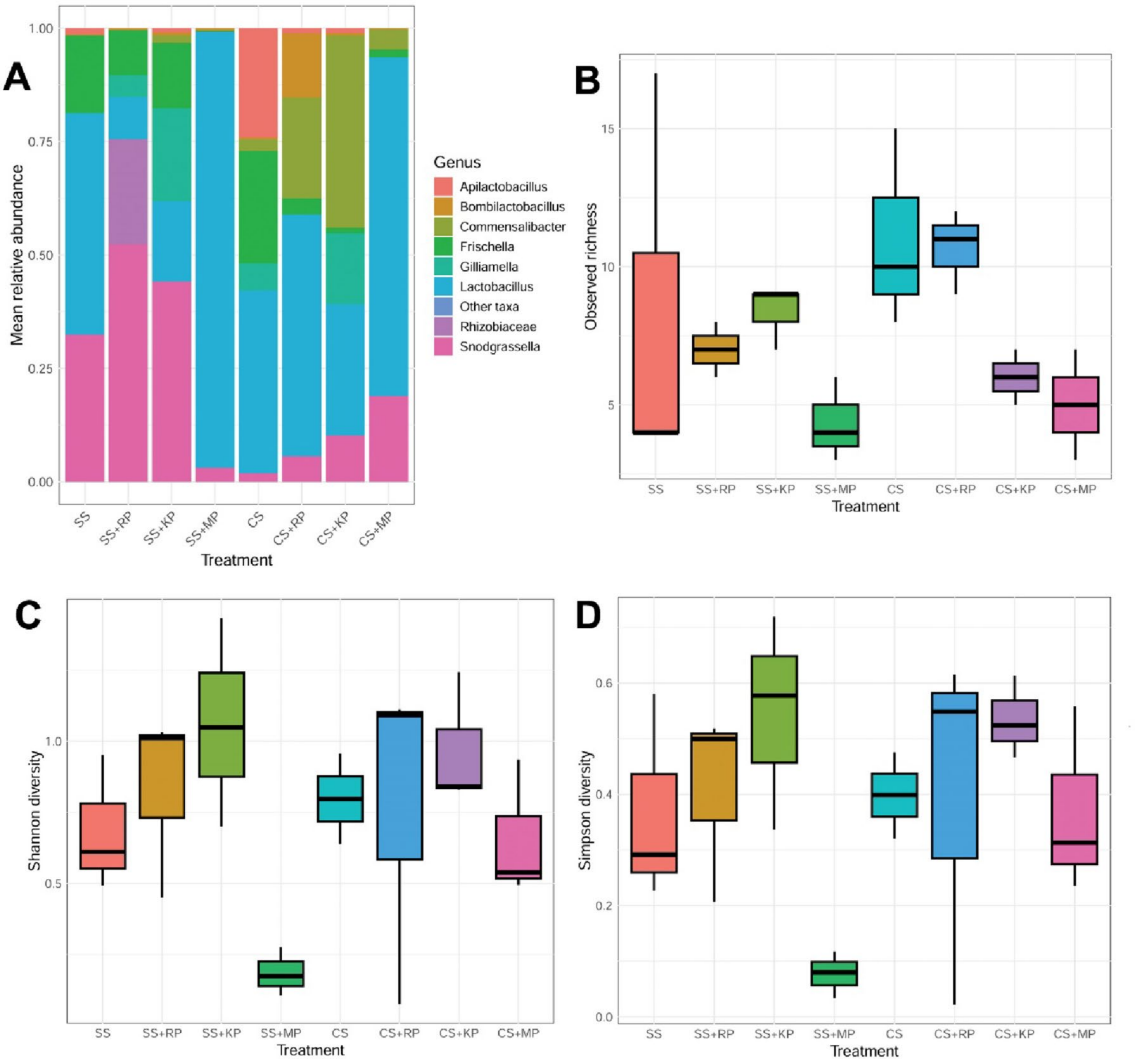
Relative abundance and alpha diversity of gut bacterial communities in 14-day-old honey bees under different dietary treatments. Bar plots showing the relative abundance of dominant gut bacterial taxa; taxa accounting for < 1% of total reads were grouped as “Other taxa”. (**A**). Observed richness across treatment groups (**B**). Shannon diversity index across treatment groups (**C**). Simpson diversity index in different treatment groups (**D**). SS: sugar syrup; CS: corn syrup; RP: rapeseed pollen; KP: kiwi pollen; MP: mixed pollen.

**Fig. 4:**
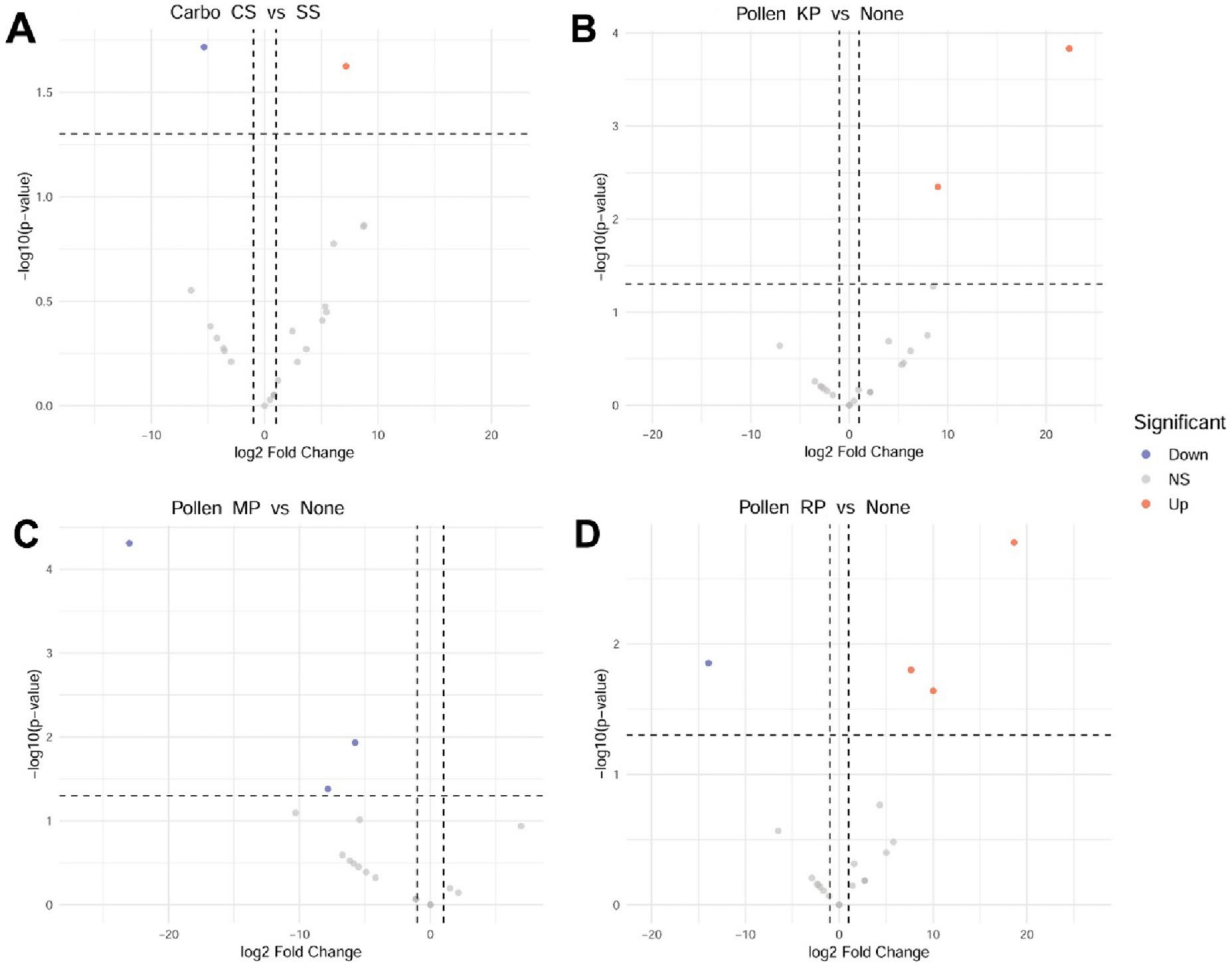
Volcano plots showing differentially abundant bacterial taxa in the honey bee gut microbiome under different carbohydrate and pollen diets. Comparison of carbohydrate sources (CS vs. SS) (**A**); Kiwi pollen (KP) vs. no pollen control (**B**); Mixed pollen (MP) vs. no pollen control (**C**); and Rape pollen (RP) vs. no pollen control (**D**). The x-axis represents the log₂ fold change in relative abundance of bacterial taxa, while the y-axis shows the − log₁₀(p-value). Vertical dashed lines indicate the fold-change threshold, and the horizontal dashed line represents the significance threshold (*p* < 0.05). Red points represent taxa significantly enriched in the treatment group, blue points represent taxa significantly depleted, and grey points represent non-significant taxa.

Differential abundance analysis using DESeq2 revealed that pollen type significantly influenced the abundance of several core bacterial taxa after false discovery rate (FDR) correction (padj < 0.05). Bees fed KP pollen showed a significantly higher abundance of members of the family Orbaceae compared with the no-pollen treatment (log₂FC = 22.36, padj = 0.0047). In contrast, the genus *Commensalibacter* was significantly less abundant in the MP treatment relative to the no-pollen group (log₂FC = − 22.99, padj = 0.0016) (Figs. 3A and 4). Comparisons among pollen treatments further revealed that KP supported higher abundances of several core gut bacteria compared with MP, including *Sondgrassella* (log₂FC = 6.69, padj = 0.027), members of the family Orbaceae (log₂FC = 35.14, padj < 0.001), *Gilliamella* (log₂FC = 14.42, padj < 0.001), and *Commensalibacter* (log₂FC = 29.23, padj < 0.001). Similarly, RP pollen resulted in significantly higher abundances of *Sondgrassella* (log₂FC = 7.35, padj = 0.013), Orbaceae (log₂FC = 31.43, padj < 0.001), and *Gilliamella* (log₂FC = 13.06, padj < 0.001) compared with the MP treatment (Figs. 3A and 4). Together, these results indicate that KP and RP pollen diets promote the proliferation of key members of the honey bee gut microbiota, whereas MP pollen is associated with reduced abundance of several core symbiotic taxa.

#### Alpha diversity

The effects of carbohydrate source and pollen type on alpha diversity indices were evaluated using aligned rank transform analysis of variance (ART ANOVA). For Observed richness, the main effect of carbohydrate source was not significant (ART ANOVA, df = 1, F = 2.47, *p* = 0.136). In contrast, pollen type significantly affected observed richness (ART ANOVA, df = 3, F = 6.01, *p* = 0.006). The interaction between carbohydrate source and pollen type was not significant (ART ANOVA, df = 3, F = 2.15, *p* = 0.134). Post hoc Tukey comparisons indicated that the MP pollen treatment had significantly lower richness than both the None treatment (*p* = 0.047) and the RP treatment (*p* = 0.0039) (Fig. 3B).

For Shannon diversity, neither carbohydrate source (ART ANOVA, df = 1, F = 0.90, *p* = 0.356) nor the interaction between carbohydrate source and pollen type (ART ANOVA, df = 3, F = 0.92, *p* = 0.451) had significant effects. However, pollen type significantly influenced Shannon diversity (ART ANOVA, df = 3, F = 3.58, *p* = 0.037). Post hoc comparisons revealed that the KP treatment showed significantly higher Shannon diversity than the MP treatment (*p* = 0.027) (Fig. 3C).

Similarly, for the Simpson diversity index, carbohydrate source did not significantly affect diversity (ART ANOVA, df = 1, F = 1.11, *p* = 0.307), and no significant interaction between carbohydrate source and pollen type was detected (ART ANOVA, df = 3, F = 0.96, *p* = 0.438). However, pollen type had a significant effect on Simpson diversity (ART ANOVA, df = 3, F = 3.64, *p* = 0.036). Tukey’s post hoc test indicated that Simpson diversity was significantly higher in the KP treatment compared with the MP treatment (*p* = 0.022) (Fig. 3D).

#### Beta diversity

**Fig. 5:**
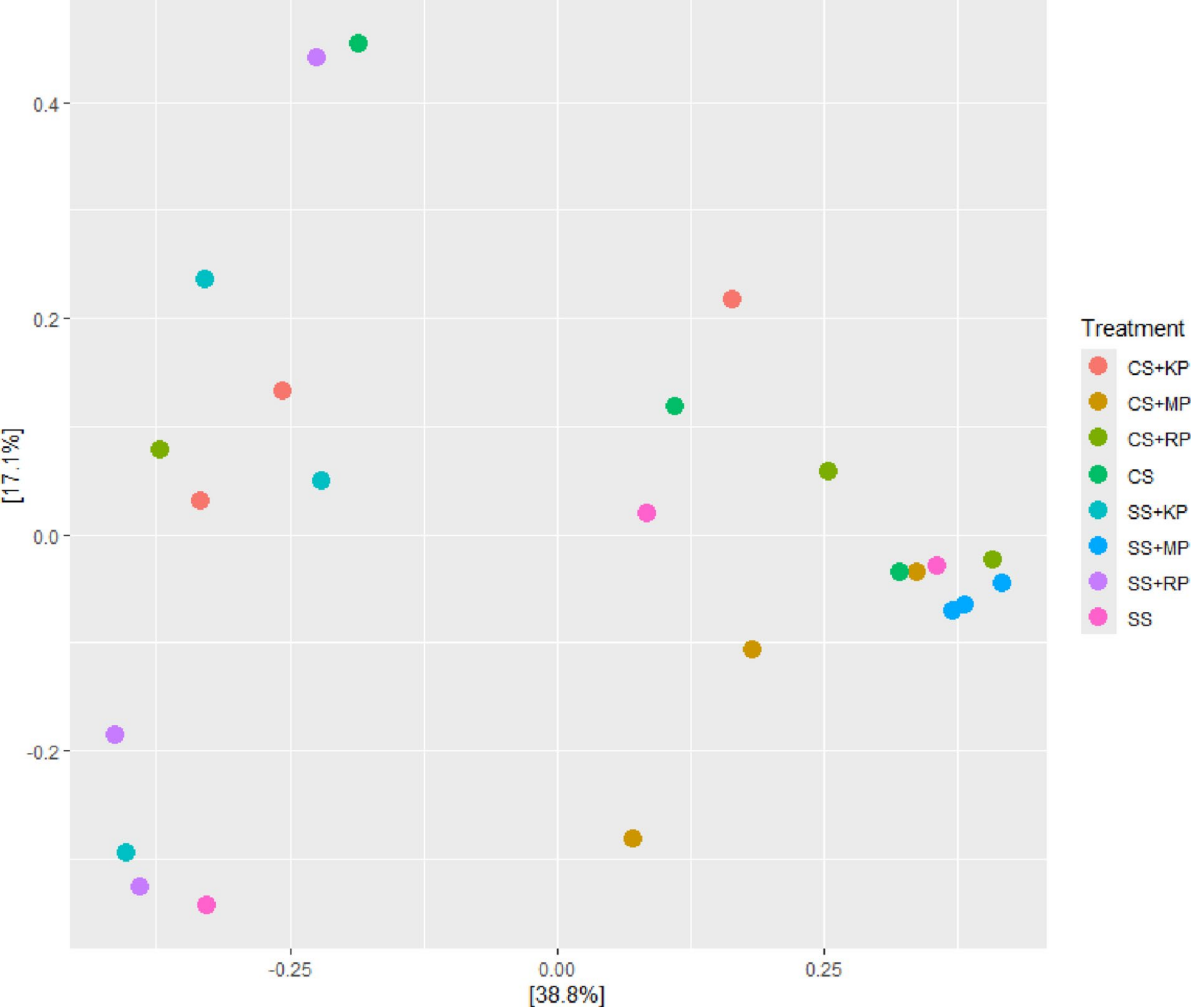
Principal coordinates analysis (PCoA) based on Bray–Curtis dissimilarity illustrating differences in gut microbiota composition among dietary treatments (SS: sugar syrup; CS: corn syrup; RP: rapeseed pollen; KP: kiwi pollen; MP: mixed pollen). Each point represents an individual replicate, and colors correspond to treatment groups. The first two axes explain 38.8% (PCoA1) and 17.1% (PCoA2) of the total variation. Differences in microbial community structure among treatments were evaluated using PERMANOVA (R² = 0.41, F = 1.62, *p* = 0.043).

Beta diversity analysis based on Bray–Curtis dissimilarity revealed differences in gut microbial community composition among dietary treatments (Fig. 5). Principal coordinates analysis (PCoA) showed that the first two axes explained 38.8% and 17.1% of the total variance in microbial community structure, respectively. Visual inspection of the ordination indicated partial clustering of samples according to treatment, suggesting that diet influenced the composition of the honey bee gut microbiota. Statistical testing using PERMANOVA confirmed that microbial community composition differed significantly among treatments (R² = 0.41, F = 1.62, *p* = 0.043), indicating that dietary treatments explained approximately 41% of the variation in gut microbial communities.

## Discussion

The results of this study offer important insights into how different combinations of carbohydrate and pollen sources influence the longevity and physiological performance of honey bee workers. Our findings emphasize the significant role that diet composition, particularly the interaction between carbohydrate type and pollen, plays in shaping honey bee survival. Bees fed with CS exhibited significantly greater longevity compared to those fed with SS, consistent with our earlier findings^42^. The CS formulation used in this experiment contained a small proportion of glucose and sucrose, but a notably high concentration of maltose (57%) along with polysaccharides (18.5%) (Table S1). These carbohydrate constituents appear to support a sustained energy supply through a staged digestion process. CS used in this study contains maltose as major components, whereas SS consists exclusively of sucrose. Maltose requires enzymatic hydrolysis to glucose prior to absorption. Therefore, differences in the relative proportions and types of sugars between CS and SS may influence the rate and temporal pattern of carbohydrate availability. Phillips^47^ reported that honey bees are capable of utilizing maltose as a carbohydrate source. Although survival varied across experiments, these findings indicate that differences in carbohydrate composition may influence honey bee performance. Together, these observations highlight the importance of carbohydrate composition in dietary formulations indicating that the inclusion of digestible disaccharides such as maltose may contribute to sustained energy availability in honey bees. Furthermore, while the type of carbohydrate plays a pivotal role, our data also show that bees fed carbohydrates alone had significantly lower survival than those fed both carbohydrates and pollen. This observation aligns with previous studies by^27^ who demonstrated that supplementing sucrose syrup with pollen improves survival rates in honey bees, indicating that a synergistic combination of energy-rich carbohydrates and protein-rich pollen not only enhances survival but may also support physiological functions essential for colony health and productivity. Although pollen consumption did not differ significantly among different treatment groups, bees fed MP showed the highest longevity and significantly larger hypopharyngeal glands, highlighting the benefits of dietary diversity. These findings align with previous studies showing that diverse pollen diets enhance bee health and immunity^27^. Those bees prefer mixed-pollen over that from sunflower or almond monocultures, linking diverse diets to improved colony health and longevity^48^. Additionally, honey bees fed with KP exhibited higher survival rates compared to those fed with RP, despite both pollen types containing similar levels of crude protein. This suggests that other macronutrients, such as fat, fiber, and nitrogen-free extract (NFE), may play a more critical role in influencing honey bee physiology and longevity (Table S2). One notable difference lies in the fat content: RP contains a significantly higher fat concentration (12.2%) compared to KP (4.5%). While lipids are vital for honey bee development, hormone synthesis, and energy storage^49^, excessive fat intake may disrupt metabolic homeostasis and reduce digestive efficiency, ultimately compromising longevity^50,51^. Also Manning^49^ demonstrated that supplementing low-lipid pollen with fatty acids, especially oleic acid at concentrations above 2%, markedly shortened honey bee lifespan in cage studies. These findings underscore the importance of balanced lipid intake and suggest that the lower fat content of KP may help avoid metabolic stress while still fulfilling essential lipid requirements. Fiber content is another key factor, RP contains more fiber (6.5%) than KP (3.1%). Because honey bees lack the enzymatic machinery to efficiently digest fibrous material, high fiber content may hinder nutrient absorption and limit the overall utility of the pollen as a food source^52^. Finally, KP offers a much higher level of NFE at 60.6%, compared to 49.5% in RP. NFE represents readily digestible carbohydrates, mainly sugars and starches, those serve as a crucial energy source for honey bee metabolic activity, immune responses, and physiological maintenance^50^. The elevated NFE content in KP may thus provide more accessible energy, supporting the observed increase in survival.

The feeding behavior data revealed that bees adjusted their carbohydrate intake based on sugar type and overall diet composition. Bees provided with SS consumed significantly more than those fed CS, likely due to the higher simplicity and digestibility of the sugars in SS. Interestingly, the highest syrup intake was observed in bees receiving only carbohydrates, suggesting that bees may increase sugar consumption to compensate for the lack of protein. Conversely, the lowest consumption occurred in bees fed with CS combined with kiwi or mixed pollen, indicating that a more nutritionally complete diet may reduce the need for excessive carbohydrate intake. Pollen consumption appeared to be less influenced by carbohydrate source. Although not statistically significant, a trend was observed where bees consumed more RP than KP, potentially due to differences in palatability or nutrient composition. One possible explanation for the higher RP intake lies in its greater fiber content. RP contains approximately twice the fiber of KP (6.5% vs. 3.1%) (Table S2), which may reduce its overall digestibility. Since honey bees lack the enzymatic machinery to efficiently break down fibrous components^52^, they may compensate by consuming more of the lower-quality pollen to meet their nutritional requirements. In contrast, the lower fiber and higher nitrogen-free extract (NFE) content in KP may have improved its digestibility and energy availability, resulting in reduced consumption despite its positive effect on longevity.

The development of hypopharyngeal glands (HPGs) in honey bee workers is closely tied to dietary quality, particularly the protein source provided through pollen^27^. Our findings show that the type of pollen significantly influences HPG size, with MP leading to the most substantial gland development, followed by RP and KP. Bees that received no pollen showed the smallest gland sizes, underscoring the critical role of protein-rich diets in supporting glandular function. The superior performance of MP likely reflects its diverse and balanced nutrient composition, providing a more complete range of amino acids, lipids, and micronutrients essential for tissue development. This aligns with earlier studies showing that pollen diversity enhances physiological outcomes, including gland size and immune function, in honey bees^27^. In addition to pollen type, the carbohydrate source also played a role in HPG development. Bees fed with CS developed larger glands than those given sucrose syrup. This suggests that beyond protein, the form and complexity of energy sources can modulate physiological traits. CS’s mixture of mono-, di-, and longer-chain carbohydrates may offer a more gradual and sustained energy release, which could support continuous protein synthesis and tissue growth. The most pronounced gland development occurred when CS was paired with MP, highlighting a synergistic effect of energy and protein sources.

When examining *vg* gene expression, bees fed MP expressed the highest levels of *vg*, while those deprived of pollen showed minimal expression. *vg* is a key biomarker of nutritional status and longevity in honey bees^53^, influencing stress resilience, immune competence, and behavioral transitions^54^. The enhanced *vg* expression in bees consuming MP is likely due to the broader range of nutrients including amino acids, vitamins and minerals that multi-floral diets may provide. Together, these findings reinforce the central role of dietary diversity and nutrient balance in supporting honey bee health. Providing access to high-quality, MP enhances glandular development and upregulates key genes like *vg*, which are crucial for longevity and colony sustainability. Although energy sources like CS can amplify these effects when paired with quality pollen, they cannot replace the need for diverse protein sources. These insights underscore the importance of floral diversity and foraging opportunities in the landscape to ensure bees receive the full spectrum of nutrients required for optimal physiological performance.

Honey bees harbor a characteristic gut microbiota composed predominantly of five core bacterial lineages, *Gilliamella*, *Snodgrassella*, *Bombilactobacillus*,* Lactobacillus*^55^, and *Bifidobacterium*, which collectively account for over 95% of the gut community^56–58^. Alongside these dominant phyllotypes, less abundant genera such as *Frischella* and *Commensalibacter* are consistently detected in honey bees^59^. Although these core taxa are consistently present in honey bees, their relative abundances can shift on factors such as season, diet, caste, age, and geographic location^60–62^. Although we acknowledge that laboratory-reared bees may not develop a fully natural gut microbiota, our goal was to minimize the confounding effects from other nectar and pollen sources, or colony interactions. This approach allowed us to isolate and accurately measure the impact of the experimental diets on gut microbiome composition, ensuring that observed differences are directly attributable to the carbohydrate and pollen treatments.

Our findings demonstrate that pollen type plays a key role in shaping the composition of the honey bee gut microbiota that aligns with previous studies^63^. The relative abundance of *Sondgrassella* and *Gilliamella* in RP and KP group was significantly higher compare to MP group. *Sondgrassella* is important components of the core microbiota and are implicated in gut epithelial integrity and pathogen defense^64,65^. However, *Gilliamella* is specialized in degrading complex carbohydrates, including pectin, derived from pollen cell walls. So it seems that single pollen source (KP, RP) may provide more consistent polysaccharide substrates that favor the proliferation of *Gilliamella* and *Sondgrassella*. Another notable shift in the gut microbiome was observed in the abundance of *Commensalibacter*, which was significantly higher in bees fed KP compared with those receiving RP and MP diets. This difference may be related to the nutritional composition of the pollen sources. KP contained a higher proportion of nitrogen-free extract (NFE), which indicates a greater availability of soluble carbohydrate fractions, which may provide substrates for carbohydrate-metabolizing bacteria such as *Commensalibacter* (Acetobacteraceae), potentially contributing to their increased abundance^31^. On the other hand, MP diet was associated with reduced alpha diversity compared with some single-pollen treatments. Specifically, MP showed lower observed richness than the no-pollen and RP treatments and lower Shannon and Simpson diversity compared with KP. This pattern may reflect the increased dominance of certain taxa, such as *Lactobacillus*, which can reduce community evenness without necessarily indicating dysbiosis. Importantly, the set of detected taxa remained broadly consistent across dietary treatments. This observation is consistent with previous studies showing that dietary interventions in honey bees typically influence the relative abundance of core gut symbionts rather than their presence or absence within the community^38,39^. Therefore, the observed differences in alpha diversity likely reflect shifts in microbial community structure driven by dietary composition rather than a loss of microbial taxa. Overall, our findings highlight that pollen type plays a key role in shaping the honey bee gut microbiota, with KP and RP diets promoting higher abundances of several core bacterial taxa compared with MP. Further colony-level studies are needed to better understand the long-term implications of carbohydrate and pollen sources on bee immunity and resilience.

## Conclusions

When comparing carbohydrate sources, CS supported longer survival and enhanced HPG development compared to SS. However, these benefits were significantly amplified when CS was paired with protein-rich and nutritionally diverse pollen source MP rather than a single source KP or RP, which further promoted HPG development and upregulated *vg* expression. Pollen sources influenced gut microbial composition. Especially pollen diversity had the most pronounced effect enriching beneficial taxa like *Lactobacillus*. Its greater abundance in the MP group may be attributed to the broader array of nutrients, particularly amino acids and micronutrients, available in diverse diets, which are often lacking in single-pollen sources. Although RP and KP contained similar protein levels, RP had higher fat and fiber, which likely contributed to larger HPGs and higher *vg* expression, whereas KP supported overall enhanced survival and greater gut bacterial diversity. Taken together, these findings highlight that honey bee health and physiological performance are not solely dependent on carbohydrate or pollen source alone but are shaped by their interactive effects. CS provides effective energy supplementation, yet its benefits are maximized when combined with diverse, protein-rich pollen. These results underscore the importance of providing bees with access to a nutritionally diverse foraging landscape, which promotes synergistic interactions between diet, gut microbiota, and physiology, ultimately supporting colony health, resilience, and long-term sustainability in both managed and natural environments.

## Materials and methods

### Assessing honey bee lifespan

This study was conducted using three healthy colonies of *Apis mellifera* derived from sister queens, maintained at the experimental apiary of Gyeongkuk National University, South Korea. All colonies had Varroa mite infestation levels below 1% and showed no signs of infectious disease. To minimize potential colony-level effects, brood frames from all three colonies were collected and incubated at 33 °C with 60% relative humidity (RH) for 24 h. Newly emerged workers were pooled and then randomly assigned to the experimental treatment groups. Honey bees were assigned to a 2 × 4 factorial experimental design consisting of two carbohydrate sources (sucrose syrup or corn syrup) and four pollen treatments: no pollen supplementation, rapeseed pollen, kiwi pollen, or a 1:1 mixture of both pollens. In total, eight treatment groups were established, each with six replicates. Each replicate consisted of 20 bees housed in cages measuring 110 × 90 × 80 mm, equipped with a fine mesh top for ventilation. Each cage was fitted with three feeding holes to hold feeders containing water, a carbohydrate solution (either sucrose syrup (SS) or corn syrup (CS)), and a protein supplement [rapeseed pollen (RP), kiwi pollen (KP), and mixed pollen (MP)] (Table S1 and S2). Protein supplements were prepared by mixing pollen with 50% sucrose syrup at a 70:30 (w/w) pollen-to-syrup ratio. The carbohydrate solutions were prepared using sucrose syrup (SS) consisted exclusively of sucrose dissolved in distilled water (50% w/w) and did not contain free glucose and corn syrup (CS) 50% (w/w) to ensure that the carbohydrate concentration was equivalent between treatments and to allow direct comparison of the effects of carbohydrate type on bee physiology and behavior. Carbohydrate resource (SS or CS) and water were also provided using two feeders placed on top of each cage. The honey bees were maintained in an incubator at 30 °C and 60% RH throughout the experiment. Daily observations were conducted to monitor mortality and food consumption rates. Mortality was recorded daily, and food consumption per bee was calculated by dividing the adjusted loss in food weight by the number of surviving bees at the point of recording. Additionally, three extra replications per treatment were included to assess physiological changes and gut microbiome composition. On the 7 and 14th day of the trial, samples were stored at -80 °C for further experiments.

### Measuring hypopharyngeal gland acinus Size

The dissection and measurement of hypopharyngeal glands (HPGs) followed the established protocols outlined by^66^. 7-day-old Worker bee heads were carefully separated and placed into individual microcentrifuge tubes. The HPGs were dissected and imaged using an Olympus SZX10 microscope (Olympus IE, Waltham, MA) equipped with an Olympus SC50 microscope camera (Olympus IE, Waltham, MA). To enhance measurement precision, five bees were selected per feeding group, with 10 acini measured per bee. The average acinus size for each treatment was calculated based on a total of 50 acini from the five bees within each group.

### RNA extraction, real-time PCR, and gene expression analysis

To investigate the effect of nutritional source on *vg* gene expression, the gut was removed from 7-day-old honey bees, and total RNA was extracted from the abdomens of five pooled individuals per replicate by using the RNeasy Mini Kit (Qiagen, Germany). Immediately after extraction, RNA quantity and purity were assessed using a Nanodrop spectrophotometer (FC-2100, Life Real, China). For each treatment, 1 µg of extracted RNA was used to synthesize complementary DNA (cDNA) using the BioFACT Reverse Transcription Kit. The synthesized cDNA samples were adjusted to a final volume of 50 µL with sterile water and stored at − 80 °C until further analysis. Real-time PCR (RT-PCR) was conducted using 100 ng of cDNA as the template, along with 10 pM of gene-specific primers (Table S3) (Fig. S1), SYBR Green Master Mix (BioFACT), and nuclease-free water, bringing the final reaction volume to 20 µL. The PCR program began with an initial denaturation at 95 °C for 15 min (one cycle), followed by 40 cycles of 95 °C for 30 s (denaturation), 52 °C for 30 s (annealing), and 72 °C for 20 s for extension. Fluorescence was measured at the end of each extension step. A dissociation step (95 °C for 15 s, 52 °C for 60 s, and 95 °C for 15 s) was performed to confirm the amplification of a single product in each PCR reaction. Actin was used as the reference gene due to its stable expression across honey bee tissues and its common use in honey bee research (Fig. S3). The relative expression of *vg* was quantified using the threshold cycle (Ct) values and analyzed using the 2^-ΔΔCt method (2^(-ΔΔct) = 2^-(Δct treatment-Δct reference))^67^. A negative control, without a cDNA template, was included to ensure the absence of contamination. All reactions were performed in triplicate for accuracy.

### Gut microbiome analysis

For gut microbiome analysis, the entire gut from five 14-day-old individuals were dissected and pooled for each biological replicate, with three replications per treatment group. Each replicate consisted of bees collected from a separate cage to ensure independence among samples. Pooling multiple individuals per replicate was performed to reduce individual variation and obtain sufficient microbial DNA for downstream sequencing. The pooled samples were placed in a 1.5 mL sterile microcentrifuge tube, and DNA was extracted using the QIAGEN DNeasy PowerSoil Kit (QIAGEN, Hilden, Germany) following the manufacturer’s instructions. The concentration of nucleic acids in each DNA sample was assessed using a Life Real spectrophotometer (Bioer Technology Co., Ltd., Zhejiang, China). Extracted DNA samples were stored at -20 °C until library preparation. A two-step PCR approach was used for library preparation, targeting the V3–V4 region of the 16 S rRNA gene. Amplification was performed using the primers 341 F (5’-CCTACGGGNGGCWGCAG-3’) and 805R (5′-GACTACHVGGGTATCTAATCC-3′), followed by sequencing on an Illumina MiSeq platform (Macrogen, Seoul, Korea). The sequences were submitted to NCBI through the project number PRJNA1299744.

The initial assessment of the quality of raw paired-end reads was performed using FastQC (Babraham Bioinformatics, Cambridge, UK). The raw sequences were imported into Qiime2, and sequence quality control, filtering, and denoising were performed using the DADA2 algorithm. Forward and reverse reads were truncated to 270 bp and 220 bp respectively, to retain high-quality sequences with a minimum Phred quality score of 20. Paired-end reads were merged, and chimeric sequences were removed during the DADA2 workflow to generate amplicon sequence variants (ASVs). Taxonomic assignment of ASVs was performed using the SILVA v132 database (Table S4).

### Statistical analysis

Statistical analyses were performed using R version 4.1.0 and SPSS Statistics version 16.0 (SPSS Inc., Chicago, USA). Kaplan–Meier survival analysis was used to evaluate the effects of different carbohydrate and pollen sources on honey bee lifespan, and pairwise comparisons were conducted using log-rank tests. The effects of carbohydrate and pollen sources on hypopharyngeal gland (HPG) size and the relative expression of the nutrition-related gene vitellogenin (*vg*) were analyzed using two-way ANOVA. For qPCR data, relative gene expression values calculated using the 2^−ΔΔCt^ method were used for statistical analysis. When the assumptions of normality were not met, factorial effects were analyzed using the Aligned Rank Transform analysis of variance (ART ANOVA) implemented in the ARTool package in R. This approach allows non-parametric analysis of factorial designs while retaining the ability to test interaction effects. When significant effects were detected, pairwise comparisons were performed using estimated marginal means with Tukey adjustment for multiple comparisons using the emmeans package. Statistical significance was determined at *p* < 0.05.

Alpha diversity metrics, including Observed richness, Shannon, Simpson, and Chao1 indices, were calculated using the phyloseq and vegan packages. Because singleton taxa were not detected in the dataset, observed and estimated richness values were nearly identical. As the Chao1 estimator relies on rare taxa, it was excluded from further analyses. Differences among treatment groups were evaluated using normality tests, Levene’s test for homogeneity of variance, and ART ANOVA followed by Tukey-adjusted pairwise comparisons when appropriate. Beta diversity was calculated using Bray–Curtis dissimilarity and visualized using principal coordinates analysis (PCoA). Statistical differences in community composition among treatments were tested using permutational multivariate analysis of variance (PERMANOVA).

Relative abundances were calculated and aggregated at the genus level, and rare taxa were pooled into an “Other taxa” category for visualization using stacked bar plots. Differential abundance analysis was performed using the DESeq2 package in R. Count data were modeled using a negative binomial generalized linear model with the design formula Carbohydrate × Pollen. Significance of coefficients was assessed using Wald tests, and p-values were adjusted for multiple testing using the Benjamini–Hochberg false discovery rate (FDR) correction.

## Supplementary Information

Below is the link to the electronic supplementary material.

Supplementary Material 1

## Data Availability

All sequence information has been deposited in the National Center for Biotechnology Information (NCBI) Sequence Read Archive (Bioproject number: PRJNA1299744).
