# Supplementary material for: Dietary pollen and carbohydrate sources influence longevity, physiology, and gut microbiota in honey bee (Apis mellifera)
Source: Sci Rep. 2026 Apr 16;16:17589. doi: 10.1038/s41598-026-48586-0 (PMC13243472; doi:10.1038/s41598-026-48586-0)
Supplement: Supplementary file 1 — Supplementary Material 1 [file 41598_2026_48586_MOESM1_ESM.docx]

**Supplementary data:**

**Interaction Effects of Carbohydrate and Pollen Sources on Honey Bee (*Apis mellifera*) Longevity, Physiology, and Gut Microbiota**

Table S1. Percentage of sugar content in sugar syrup and corn syrup

|  | Fructose | Glucose | sucrose | Maltose | Moisture | Others |
| --- | --- | --- | --- | --- | --- | --- |
| Corn syrup | 0.00 | 0.79 | 0.83 | 57.08 | 22.8 | 18.5 |
| White sugar 50% | 0.00 | 0.00 | 49.9 | ND | 50 | 0.01 |

Table S2. Nutritional Composition of Rapeseed and Kiwi Pollen.

|  | Rapeseed Pollen  (Ghosh and Jung., 2020) | Kiwi Pollen  (Ghosh and Jung., 2017) |
| --- | --- | --- |
| Moisture | 4.5 | 13.8 ± 0.01 |
| Protein | 26.8 ± 0.07 | 26.5 ± 0.2 |
| Fat | 12.2 ± 0.04 | 4.5 ± 0.02 |
| Fiber | 6.5 ± 0.08 | 3.1 ± 0.3 |
| Ash | 5.3 ± 0.03 | 5.2 ± 0.04 |
| NFE | 49.5 ± 0.03 | 60.6 ± 0.1 |

Table S3. Primers for genes expression analysis and associated melting temperatures (Tm).

| Target genes | Gene description | Primer sequence forward and reverse | Tm (°C) | Reference |
| --- | --- | --- | --- | --- |
| *Vg* | Vitellogenin | TCAAGGATTGCGTGGAGGAC  AAAGGGCTATAAGGGCGTCG | 62.9  61.8 | Najarpoor et al., 2025 |
| *Act* | Actin | TCCGAAGGCCAATCGTGAA  CCGGTAGTACGTCCAGAAGC | 62.5  62.5 | Najarpoor et al., 2025 |


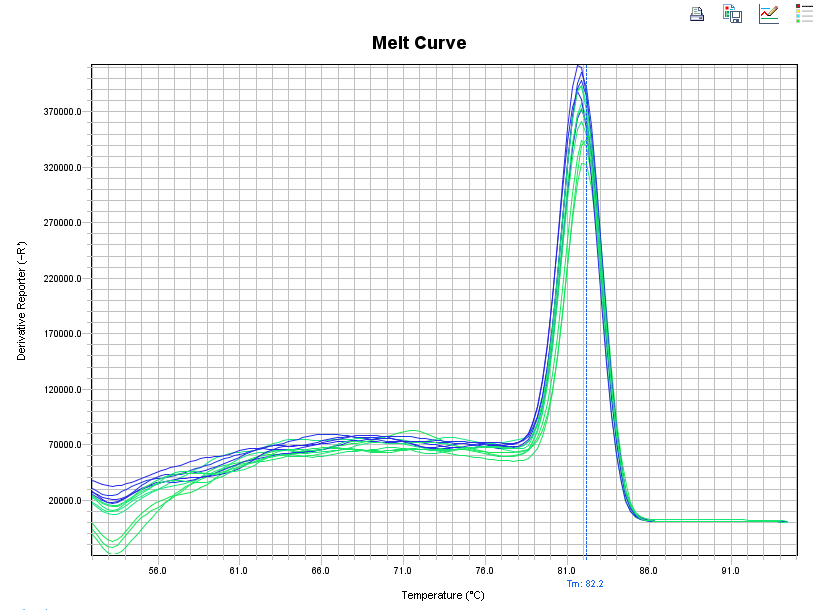

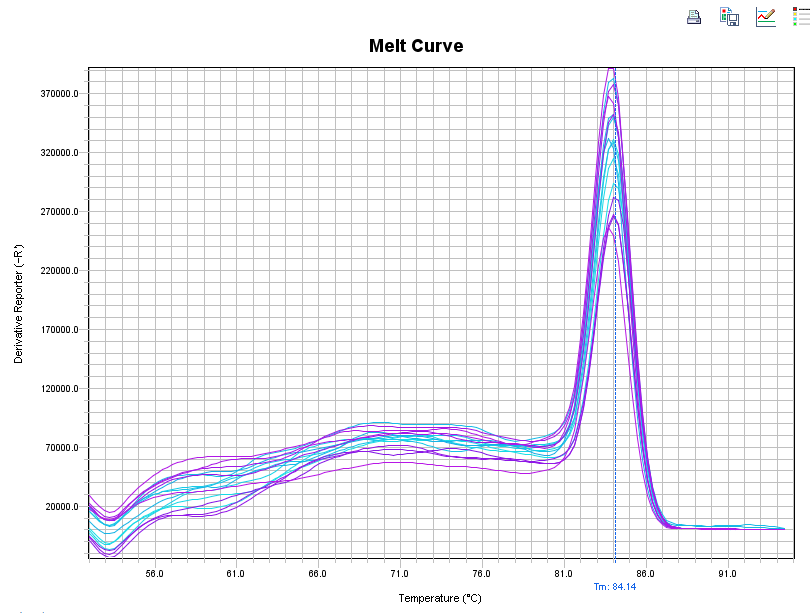


Figure S1. Melt curve analysis of qPCR amplicons for actin (left) and vg (right) showing a single sharp peak at ~82.2°C and ~84.1°C respectively, indicating specific amplification without primer-dimer formation or non-specific products.

Table S4. Relative abundance (%) of bacterial genera in 14-day-old honey bee gut samples across different treatments. SS: sugar syrup; CS: corn syrup; RP: rapeseed pollen; KP: kiwi pollen; MP: mixed pollen.

| Class | Phylum | Order | Family | Genus | SS | SS+RP | SS+KP | SS+MP | CS | CS+RP | CS+KP | | | CS+MP | |
| --- | --- | --- | --- | --- | --- | --- | --- | --- | --- | --- | --- | --- | --- | --- | --- |
| Actinobacteria | Actinobacteria | Bifidobacteriales | Bifidobacteriaceae |  | 0.00 | 0.00 | 0.03 | 0.00 | 0.00 | 0.00 | | 0.00 | 0.00 | |  |
| Actinobacteria | Actinobacteria | Bifidobacteriales | Bifidobacteriaceae | *Bifidobacterium* | 0.00 | 0.2 ± 0.1 | 0.1 ± 0.08 | 0.2 ± 0.2 | 0.1 ± 0.1 | 3.8 ± 3.6 | | 0.00 | 0.1 ± 0.1 | |  |
| Actinobacteria | Actinobacteria | Propionibacteriales | Nocardioidaceae | *Nocardioides* | 0.06 ± 0.1 | 0.00 | 0.00 | 0.00 | 0.00 | 0.00 | | 0.00 | 0.00 | |  |
| Bacteroidota | Bacteroidia | Cytophagales | Cyclobacteriaceae | *Mongoliitalea* | 0.06 ± 0.07 | 0.00 | 0.00 | 0.00 | 0.00 | 0.00 | | 0.00 | 0.00 | |  |
| Bacteroidota | Bacteroidia | Flavobacteriales | Flavobacteriaceae | *Flavobacterium* | 0.00 | 0.005 ± 0.01 | 0.00 | 0.00 | 0.00 | 0.00 | | 0.00 | 0.00 | |  |
| Firmicutes | Bacilli | Lactobacillales | Lactobacillaceae | *Bombilactobacillus* | 0.1 ± 0.1 | 0.3 ± 0.2 | 0.5 0.2 | 0.5 ± 0.3 | 0.3 ± 0.2 | 0.1 ± 0.5 | | 0.5 ± 0.1 | 0.2 ± 0.1 | |  |
| Firmicutes | Bacilli | Lactobacillales | Lactobacillaceae | *Lactobacillus* | 48.3 ± 20.8 | 9.1 ± 10.8 | 17.5 ± 5.8 | 95.9 ± 1.2 | 39.6 ± 21.6 | 51.1 ± 26.3 | | 28.9 ± 13.4 | 74.5 ± 9.4 | |  |
| Firmicutes | Bacilli | Lactobacillales | Lactobacillaceae | *Apilactobacillus* | 1.3 ± 1.3 | 0 | 0.9 ± 0.9 | 0.00 | 23.7 ± 23 | 1.1 ± 0.6 | | 1 ± 1 | 0.00 | |  |
| Firmicutes | Bacilli | Lactobacillales | Lactobacillaceae | *Holzapfelia* | 0.00 | 0.00 | 0.00 | 0.00 | 0.005 ± 0.01 | 0.00 | | 0.00 | 0.00 | |  |
| Firmicutes | Clostridia | Clostridiales | Clostridiaceae | *Tyzzerella* | 0.09 ± 1.1 | 0.00 | 0.00 | 0.00 | 0.00 | 0.06 ± 0.03 | | 0.00 | 0.00 | |  |
| Firmicutes | Bacilli | Bacillales | Bacillaceae | *Bacillus* | 0.2 ± 0.1 | 0.00 | 0.00 | 0.00 | 0.08 ± 0.09 | 0.00 | | 0.00 | 0.00 | |  |
| Firmicutes | Bacilli | Paenibacillales | Paenibacillaceae | *Paenibacillus* | 0.00 | 0.00 | 0.2 ± 0.2 | 0.00 | 0.00 | 0.00 | | 0.00 | 0.00 | |  |
| Firmicutes | Bacilli | Lactobacillales | Streptococcaceae |  | 0.00 | 0.00 | 0.00 | 0.00 | 0.00 | 0.01 ± 0.02 | | 0.00 | 0.00 | |  |
| Firmicutes | Bacilli | Staphylococcales | Staphylococcaceae | *Staphylococcus* | 0.00 | 0.00 | 0.00 | 0.00 | 0.01 ± 0.01 | 0.00 | | 0.00 | 0.00 | |  |
| Firmicutes | Clostridia | Lachnospirales | Lachnospiraceae |  | 0.00 | 0.00 | 0.00 | 0.00 | 0.1 ± 0.01 | 0.00 | | 0.00 | 0.00 | |  |
| Proteobacteria | Gammaproteobacteria | Pseudomonadales | Pseudomonadaceae | *Pseudomonas* | 0.00 | 0.00 | 0.00 | 0.00 | 0.1 ± 0.1 | 0.009 ± 0.01 | | 0.00 | 0.00 | |  |
| Proteobacteria | Gammaproteobacteria | Burkholderiales | Neisseriaceae | *Snodgrassella* | 32.1 ± 25.2 | 51.5 ± 26.6 | 43.9 ± 18.3 | 3.03 ± 1.3 | 1.8 ± 1.4 | 5.3 ± 5.2 | | 10.2 ± 5.2 | 18.8 ± 9.3 | |  |
| Proteobacteria | Alphaproteobacteria | Rhizobiales | Rhizobiaceae |  | 0.07 ± 0.08 | 22.9 ± 22.9` | 0.00 | 0.00 | 0.00 | 0.00 | | 0.00 | 0.00 | |  |
| Proteobacteria | Gammaproteobacteria | Enterobacterales | Enterobacteriaceae | *Orbaceae* | 0.00 | 0.00 | 0.1 ± 0.1 | 0.00 | 0.00 | 0 | | 0.02 ± 0.03 | 0.00 | |  |
| Proteobacteria | Gammaproteobacteria | Enterobacterales | Enterobacteriaceae | *Escherichia* | 0.04 ± 0.08 | 0 | 0.00 | 0.00 | 0.00 | 0.00 | | 0.00 | 0.00 | |  |
| Proteobacteria | Gammaproteobacteria | Enterobacterales | Enterobacteriaceae | *Frischella* | 16.8 ± 16.4 | 9.7 ± 2.4 | 14.2 ± 11.6 | 0.1 ± 0.1 | 24.4 ± 24.3 | 3.3 ± 3.4 | | 1.1 ± 0.9 | 1.8 ± 1.8 | |  |
| Proteobacteria | Alphaproteobacteria | Caulobacterales | Caulobacteraceae | *Caulobacter* | 0.00 | 0.00 | 0.00 | 0.00 | 0.00 | 0.01 ± 0.01 | | 0.00 | 0.01 ± 0.01 | |  |
| Proteobacteria | Gammaproteobacteria | Pseudomonadales | Moraxellaceae | *Acinetobacter* | 0.00 | 0.00 | 0.00 | 0.00 | 0.06 | 0.00 | | 0.00 | 0.00 | |  |
| Proteobacteria | Gammaproteobacteria | Burkholderiales | Rhodocyclaceae | *Methyloversatilis* | 0.05 ± 0.05 | 0.00 | 0.00 | 0.00 | 0.06 ± 0.07 | 0.006 ± 0.01 | | 0.00 | 0.00 | |  |
| Proteobacteria | Gammaproteobacteria | Enterobacterales | Pasteurellaceae | *Haemophilus* | 0.00 | 0.00 | 0.02 | 0.00 | 0.00 | 0.007 ± 0.01 | | 0.00 | 0.00 | |  |
| Proteobacteria | Gammaproteobacteria | Enterobacterales | Orbaceae |  | 0.00 | 0.00 | 0.00 | 0.00 | 0.01 ± 0.01 | 0.00 | | 0.00 | 0.00 | |  |
| Proteobacteria | Gammaproteobacteria | Enterobacterales | Orbaceae | *Gilliamella* | 0.2 ± 0.1 | 4.7 ± 3.1 | 20.4 ± 18.4 | 0.01 ± 0.01 | 5.8 ± 5.1 | 0.02 ± 0.01 | | 15.6 ± 12.7 | 0.00 | |  |
| Proteobacteria | Alphaproteobacteria | Sphingomonadales | Sphingomonadaceae | *Sphingobium* | 0.06 ± 0.06 | 0.00 | 0.00 | 0.05 ± 0.06 | 0.00 | 0.00 | | 0.00 | 0.00 | |  |
| Proteobacteria | Gammaproteobacteria | Enterobacterales | Enterobacteriaceae | *Aquabacterium* | 0.07 ± 0.07 | 0.00 | 0.00 | 0.00 | 0.2 ± 0.2 | 0.00 | | 0.00 | 0.00 | |  |
| Proteobacteria | Alphaproteobacteria | Sphingomonadales | Sphingomonadaceae | *Novosphingobium* | 0.00 | 0.00 | 0.009 ± 0.01 | 0.00 | 0.00 | 0.00 | | 0.00 | 0.00 | |  |
| Proteobacteria | Alphaproteobacteria | Sphingomonadales | Sphingomonadaceae |  | 0.06 ± 0.07 | 0.00 | 0.00 | 0.00 | 0.2 ± 0.2 | 0.03 ± 0.03 | | 0.00 | 0.00 | |  |
| Proteobacteria | Alphaproteobacteria | Acetobacterales | Acetobacteraceae |  | 0.00 | 0.00 | 0.00 | 0.00 | 0 | 0.005 ± 0.01 | | 0.00 | 0.00 | |  |
| Proteobacteria | Gammaproteobacteria | Burkholderiales | Neisseriaceae |  | 0.00 | 0.01 ± 0.01 | 0 | 0.00 | 0.00 | 0.00 | | 0.00 | 0.00 | |  |
| Proteobacteria | Gammaproteobacteria | Enterobacterales | Acetobacteraceae | *Commensalibacter* | 0.07 | 0.00 | 1.6 ± 1.6 | 0.00 | 2.5 ± 2.5 | 21.3 ± 21 | | 42.4 ± 21.5 | 4.4 ± 2.5 | |  |
| Proteobacteria | Gammaproteobacteria | Enterobacterales | Comamonadaceae |  | 0.00 | 0.00 | 0.00 | 0 | 0.1 | 0.00 | | 0.00 | 0.00 | |  |
| Proteobacteria | Alphaproteobacteria | Rhizobiales | Devosiaceae | *Devosia* | 0.005 ± 0.01 | 0.00 | 0.00 | 0.00 | 0.1 ± 0.1 | 0.00 | | 0.00 | 0.00 | |  |
| Proteobacteria | Gammaproteobacteria | Burkholderiales | Alcaligenaceae | *Alcaligenes* | 0.00 | 0.00 | 0.00 | 0.00 | 0.08 ± 0.08 | 0.00 | | 0.00 | 0.00 | |  |
| Proteobacteria | Alphaproteobacteria | Rhizobiales | Rhizobiaceae | *Rhizobium* | 0.00 | 0.00 | 0.00 | 0.00 | 0.05 ± 0.05 | 0.00 | | 0.00 | 0.00 | |  |
